# 1422. Surveillance for fungi in hospital environments housing high-risk hosts using culture and culture-independent methods

**DOI:** 10.1093/ofid/ofad500.1259

**Published:** 2023-11-27

**Authors:** Cornelius J Clancy, Shaoji Cheng, Binghua Hao, Eileen Driscoll, Giuseppe Fleres, Katherine Buser, Alexander Sundermann, Ashley Ayres, Graham M Snyder, M Hong Nguyen

**Affiliations:** University of Pittsburgh, Pittsburgh, Pennsylvania; University of Pittsburgh, Pittsburgh, Pennsylvania; University of Pittsburgh Medical Center, Pittsburgh, Pennsylvania; University of Pittsburgh, Pittsburgh, Pennsylvania; University of Pittsburgh, Pittsburgh, Pennsylvania; University of Pittsburgh, Pittsburgh, Pennsylvania; University of Pittsburgh, Pittsburgh, Pennsylvania; UPMC, Pittsburgh, Pennsylvania; University of Pittsburgh, Pittsburgh, Pennsylvania; University of Pittsburgh School of Medicine, Pittsburgh, Pennsylvania

## Abstract

**Background:**

Nosocomial outbreaks of fungal infections are increasingly reported. There are scant data on fungal burdens in hospital environments. Standardized surveillance methods are lacking.

**Figure:**
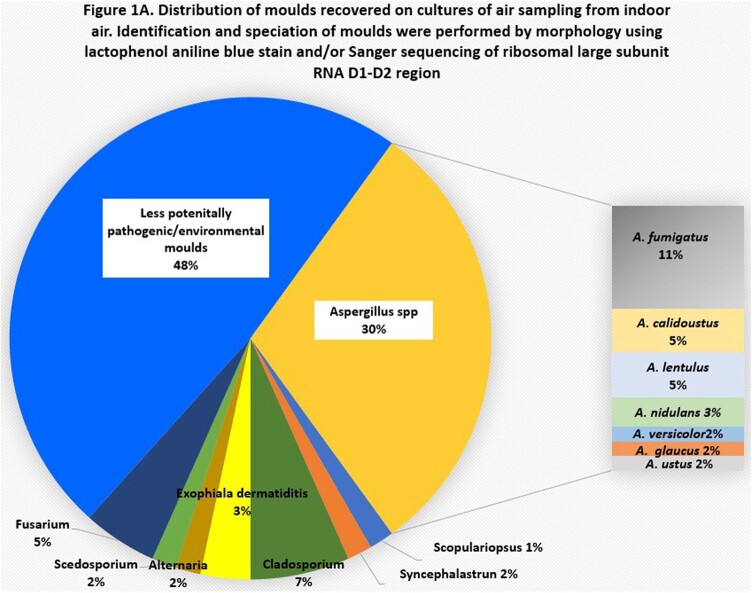

**Figure:**
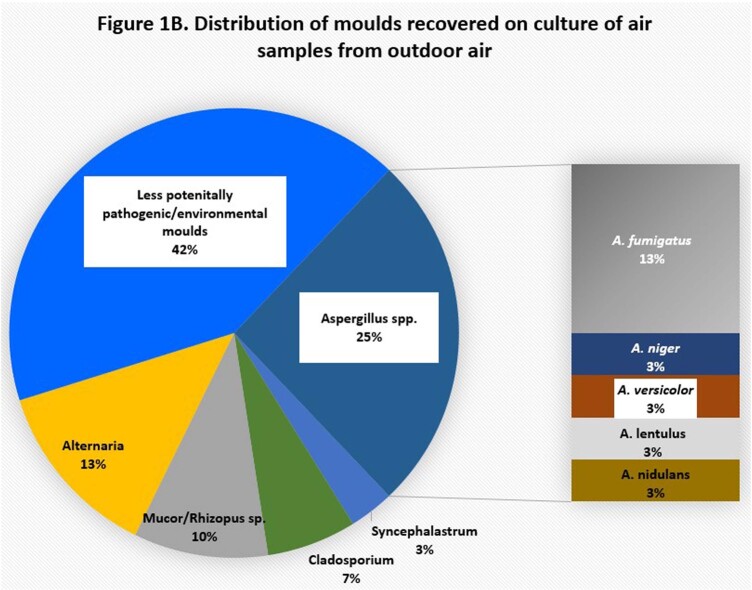

**Methods:**

We developed culture and culture-independent (real-time PCR, reference to standard curve) methods for measuring airborne fungal burdens using SAS Super 100 and SASS 3100 Dry Air samplers. We performed serial surveillance in 7 units with high-risk, immunosuppressed patients in 2 hospitals and outside.

**Figure:**
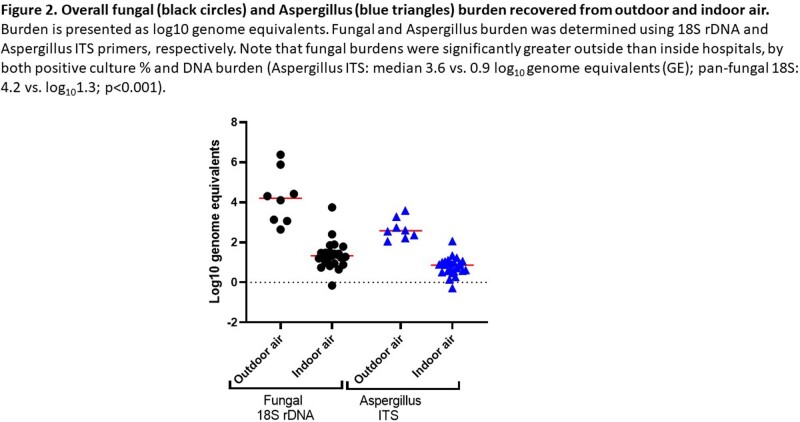

**Results:**

Methods were optimized for multiple variables, including volume, duration, flow of air sampling; culturing techniques (direct impact plates, cultures at different stages of filter processing); filter DNA recovery (extraction protocols, stage of filter processing); PCR (targets, primers, PCR parameters). To date, we have surveyed each unit ≥4 times. Pathogenic moulds were cultured in >50% of outdoor and indoor air, including azole-resistant *Aspergillus* species [Figures 1A-B]. Fungal burdens were significantly greater outside than inside hospitals, by both positive culture % and DNA burden [Figure 2]. There was no correlation between outside and inside genome equivalents (GEs; R^2^< 0.06), nor were there significant differences in GEs within a given unit (nurses stations, patient rooms; all p >0.1). GEs were greater in culture-positive than culture-negative samples (Aspergillus, 2.4 vs. 1.5 log_10_; pan-fungal, 2.8 vs. 1.5 log_10_GE; p< 0.0001; best results with sonicated filters). Direct impact cultures were less sensitive than filter cultures.

**Conclusion:**

We developed standardized methods for sampling airborne fungi in hospitals that yield reproducible results. Fungal burdens were significantly lower in hospitals than those immediately outside, but fungal DNA and viable Aspergillus and other moulds were commonly recovered from units housing high-risk patients. Outside fungal burdens could not be used to estimate relative burdens within hospitals. Direct impact cultures, commonly used in surveillance, lacked sensitivity for detecting viable fungi and did not correlate with DNA burdens. We are optimizing direct metagenomic sequencing from airborne samples. We will assess correlations between environmental surveillance data and nosocomial fungal infections.

**Disclosures:**

**Alexander Sundermann, DrPH, CIC, FAPIC**, OpGen: Honoraria **Graham M. Snyder, MD, SM**, Infectious Diseases Connect: Advisor/Consultant

